# Transcript Analysis of Heat Shock Protein 72 in Vitrified 2-Cell Mouse Embryos and Subsequent *In Vitro* Development

**Published:** 2013-11-20

**Authors:** Afrooz Habibi, Naser Farrokhi, Joaquim Fernando Moreira da Silva, Ahmad Hosseini

**Affiliations:** 1 Department of Anatomical Sciences, International Branch, Shiraz University of Medical Sciences, Kish Island, Iran; 2 Department of Agronomy and Plant Breeding, Faculty of Agriculture, Shahrood University of Technology, Shahrood, Iran; 3 Animal Reproduction, Department of Agrarian Sciences, University of the Azores, Angra do Heroı´smo, Portugal; 4 Cellular and Molecular Biology Researcher Center, Shahid Beheshti University of Medical Sciences and Health Services, Tehran, Iran

**Keywords:** Murine, Preimplantation Embryo Development, Quantitative PCR, Vitrification

## Abstract

**Objective:**

The aim of the study was to compare the effects of two different concentrations of cryoprotectants by cryotopvitrification on survival, developmental capacity and *Heat shock protein 72 (Hsp72)* expression of two-cell mouse embryos.

**Materials and Methods:**

In this experimental study, transcript analysis of *Hsp72* gene was performed on non-vitrified and vitrified 2-cell mouse embryos via a nested quantitative polymerase chain reaction (nqPCR) subsequent to normalization with Hprt1 as the reference gene. The different cryoprotectant combinations were 15% (vit1:7.5% of each ethylene glycol (EG) and dimethyl sulfoxide (DMSO), 30% (vit_2_:15% EG + 15% DMSO) and control group with no cryoprotectants. Vitrified and fresh 2-cell embryos were cultured to obtain cleavage and blastocyst formation rates. The results were analyzed via one-way analysis of variance and the mean values were compared with least significant difference (LSD) (p< 0.05).

**Results:**

The relative expression of *Hsp72* in vit_2_ (30% v/v) was significantly higher than vit1 (15% v/v). Survival rates were the same for both vitrification treatments and significantly lower than the control group. Cleavage and blastocyst rates in vit1 were significantly higher than vit_2_ while those in two vitrified groups were significantly lower than the control group.

**Conclusion:**

Our developmental data demonstrated that vit1 treatment (7.5% EG and 7.5% DMSO) was more efficient than vit_2_ (15% EG and 15% DMSO) in mouse embryos. The cryotopvitrification with two concentrations of cryoprotectants caused the relative changes of *Hsp72* transcript level, but the stability of the gene in vit1 was significantly higher than vit_2_ and closer to the fresh 2-cell embryos.

## Introduction

In assisted reproduction, embryo cryopreservation has proven to be a powerful tool with applications in bioscience, agriculture and medicine (1). It has been demonstrated that ovarian hyperstimulation syndrome can be decreased via embryo cryopreservation.

Additionally, it has been noted that embryo cryopreservation can reduce the occurrence of multiple pregnancies and preserve the fertility of cancer patients (2-4). However, little is known about its molecular impacts on embryos and the future newborne. Hence, considering molecular changes that may occur during and subsequent to embryo cryopreservation would provide a better picturefor decision making and managing probable undesirable outcomes. Evaluating the alterations of particular transcripts that may occur upon cryopreservation or global analysis of transcripts would be the first step towards answering some of the raised questions.

Cryopreservation of embryos usually can be performed either through slow freezing or vitrification. Commonly, a combination of high concentrations of cryoprotectants (typically dimethyl sulfoxide (DMSO) and ethylene glycol (EG) in addition to dehydrating agents such as sucrose or sorbitol have been used in vitrification. Extremely fast cooling of embryos via avoiding ice crystal formation allows vitrification to occur with minimum damage to the cells (4,5). However, use of high concentrations of cryoprotectants, which are often toxic to the cells may raise some questions regarding the safety issues of this technique (6).

Meanwhile, alterations of vitrification methodology can provide insights to reduce some of its drawbacks. These modifications can be performed either via increasing the cooling rate, a method known as ultra-rapid vitrification (7), or through reducing the vitrification solution volume. It has been suggested that even cells in pure water (without cryoprotectant) can be vitrified if the cooling rate is sufficiently high (8).

Ultra-rapid vitrification methods employ the use of miniature devices, allowing to freeze cells in sub-microlitre volumes (4). Electron microscope grids (9), open-pulled straws (10), cryoloops (11), microdrops (12,13), cryotops (14), solid surface vitrification (15), nylon mesh (16) and cryotip (17) are amongst successful tools developed in recent years. The approach that minimizes the volume of vitrification solution is the cryotop (14). Cryotop allows loading of very small volume as little as 0.1 μl, improving the cooling rate to increase to 23,000 ˚C/minute. Consequently, higher cooling rate allows to useless concentrated solutions and eventually lessening any potentially toxic effects (17,18).

Expression of many genes including Heat shock protein (HSP) family, as its name indicates, is mainly affected in response to the changes in temperature (19). It was previously reported that the expression of *Hsp72/Hsp73* is increased at the 2-cell stage (20). Accordingly, 2-cell mouse embryos were cryopreserved in the presence of two concentrations of cryoprotectants (30 and 15%) and subsequent changes of *Hsp72* and Hprt1 (housekeeping gene) were analyzed upon thawing. Cryotop was the instrument of choice for vitrification. Vitrified and fresh 2-cell embryos were cultured to obtain cleavage and blastocyst formation rates. The aim of the study was to compare the effects of two different concentrations of cryoprotectants by cryotop vitrification on survival, developmental capacity and *heat shock protein 72 (Hsp72)* expression of two-cell mouse embryos.

## Materials and Methods

This was an experimental study. This project was approved by the Ethics Committee of Shahid Beheshti University of Medical Sciences in 2009. All chemicals were purchased from Sigma Chemical (St Louis, MO, USA) unless it has been stated otherwise.

CD1 (ICR) female mice aged 8-10 weeks and male mice aged 10-12 weeks (Lisbon University, Portugal) were housed in polycarbonate cages (12 hours light/dark, 22 ± 2˚C), and were fed with standard food and fresh water. In all procedures, mice were handled according to the rules stipulated by the Animal Care in Portugal.

### Preparation of 2-cell embryo

Female mice were super ovulated by intraperitoneal injection of 10 IU pregnant mare serum gonadotropin (PMSG), followed by 10 IU of human chorionic gonadotropin (hCG) with a 48 hours interval. Female and male mice (1:1) were mated and checked for vaginal plugs the next morning. The plug-positive female mice were sacrificed by cervical dislocation at 48 hours post-hCG injection (4,21), and 2-cell embryos were collected by flushing oviducts into potassium simplex optimized medium (KSOM^+AA^) (Millipore, MA, USA) supplemented with 4 mg/ml bovine serum albumin (BSA) and 20 mMN-2-Hydroxyethylpiperazine- N'-2-Ethanesulfonic Acid (Hepes) buffer (5,22).

### Study groups

The embryos were vitrified in two different concentrations of cryoprotectants by Cryotop and the changes of *Hsp72* expression, survival, cleavage and blastocyst formation rates in vitrified and nonvitrified groups were assessed. The embryos from the mice sacrificed on each day were collected and then divided into two main groups, vitrified and control (non-vitrified) groups: the vitrified group was divided into two subgroups vit1 (15% v/v: 7.5% DMSO+7.5% EG) and vit_2_ (30% v/v: 15% DMSO+15% EG). Finally, 195 embryos of vitrified and control groups were evaluated for survival, cleavage and blastocyst rates. 200 embryos were assessed for expression of *Hsp72* and Hprt1 as the reference gene (23,24).

For gene expression, each embryo pool containing 10 embryos was stored at -80˚C in a minimum volume (2 μl) of RNase free water (23). Experiments in each series were repeated at least three times.

### Vitrification and thawing solutions

As the basal medium or washing solution (WS), modified Dulbecco’s phosphate-buffered saline solution containing 10% (v/v) fetal bovine serum (GIBCO, CA, USA) was used. The equilibration solution contained 7.5% (v/v) EG and 7.5% (v/v) DMSO in basal medium.

There were two vitrification solutions (VS) for two vitrified groups, VS1: 7.5% (v/v) EG, 7.5% (v/v) DMSO and 0.5 mol/l sucrose in basal medium and VS2: 15% (v/v) EG, 15% (v/v) DMSO and 0.5 mol/l sucrose in basal medium. Thawing solution contained 0.5 M sucrose and diluent solutions (D1, D2, D3, D4, and D5) contained 0.4, 0.3, 0.2, 0.1 and 0.05 M sucrose in basal medium, respectively.

### Vitrification and thawing

Two concentrations of vitrification solutions were used to vitrify the mouse 2-cell embryos using Cryotop. Embryos of vit1 and vit_2_ groups were placed in three droplets of equilibration solution for 1 minute total for all of the drops at 25˚C. Subsequently, embryos were transferred into vitrification solution VS1 and VS2 respectively for less than 30 seconds. Embryos (6) were moved on the Cryotop (<1 μl vitrification solution) and the Cryotop was immediately submerged in filter-sterilized liquid nitrogen and kept for at least 7 days.

Samples were thawed by plunging the Cryotop into 1 ml of thawing solution at 37˚C for 1 minute. Rehydration and gradual removal of cryoprotectants were performed in D1, D2, D3, D4 and D5 for 3 minutes at every step. Thawed embryos were then washed three times in basal medium (Dulbecco’s Dulbecco’s phosphate-buffered saline solution) for 5 minutes at 25˚C.

### Definition of morphological surviv

Embryos were defined “morphologically survived”, if the embryos possessed an intact zona pellucida, blastomeres and refractive cytoplasm (25,26). Following the thawing and cryoprotectant removal steps, embryos in 100 μl of sterilized KSOM^+AA^ (Millipore, MA, USA) supplemented with 4 mg/ml BSA were incubated under mineral oil with the availability of 5% (v/v) CO_2_, 5% (v/v) O_2_, and 90% (v/v) N2 for 1 hour at 37˚C.

The validity of morphological classification was confirmed by vital staining with 0.4% sterilized trypan blue solution, a plasma membrane specific dye, in Hanks’ balanced salt solution (HBSS) (27,28). The embryos were examined under an inverted micromanipulation microscope (Eppendorf, NY, USA). The dead cells were stained dark blue by trypan blue but viable cells were able to repel the dye and were not stained. They were counted and recorded as survival rates. Visually dead embryos were discarded, and the morphologically intact embryos were cultured and the gene expression pattern was analyzed.

### Embryo culture

The survived embryos in control, vit1 and vit_2_ groups were cultured in 20 μl droplets of KSOM^+AA^ supplemented with 4 mg/ml BSA under mineral oil at 37˚C in an atmosphere of 5% CO_2_, 5% O_2_ and 90% N2 to develop into blastocysts. Embryos were assessed daily to record cleavage and blastocyst formation rates for 4 days.

### Gene expression

The relative quantification of gene transcripts was carried out by real-time PCR. Super Script™ III Platinum® Cells Direct Two-Step Quantitative reverse transcriptase PCR (qRT-PCR) Kit with SYBR® Green (Invitrogen, CA, USA) was used to carry out cDNA synthesis and PCR.

### Reverse transcription reaction

Embryos were lysed in 1 μl lysis enhancer and 10 μl resuspension buffer for every PCR tube, which were incubated at 75˚C for 10 minutes in a Thermal Cycler (Applied Biosystems 9700, CA, USA). To degrade any contaminating DNA, the cell lysates were treated with 5 μ1DNase I and 1.6 μl DNase I buffer (10×) at 25˚C for 5 minutes. The embryos were treated with 4 μl of 25-mM EDTA and incubated at 70˚C for 10 minutes. For first- Strand cDNA Synthesis, 20 μl 2× RT Reaction Mix and 2 μl RT Enzyme Mix were added to each tube which was then incubated at 25, 50 and 85˚C for 10, 20 and 5 minutes, respectively.

### Nested quantitative polymerase chain reaction

Sometimes the expressions of some genes are very low, which makes the absolute quantification near to impossible. In such cases a prior polymerase chain reaction (PCR) amplification is required to boost the template level for the following quantification via Real-Time PCR, a technique called "nested quantitative PCR" or nqPCR for short (29,30). It is noteworthy to mention that the use of PCR amplicons instead of cDNA for the absolute quantification is not as accurate. However in places where the relative quantification serves the purpose, nqPCR provides enough accuracy. Additionally, considering the number of cells or the quantity of RNA that is used for cDNA synthesis, the expression level can be calculated.

The Primer pairs for each gene were designed, synthesized and validated by Molecular Diagnostic Companies (MDC, Burgess Hill, UK). The primer sequences, annealing temperatures and Gen Bank accession numbers are provided in table 1.

**Table 1:** Primers and conditions used for quantification of gene expression by real-time PCR


Gene symbol	Gen Bank accession	Sense primer (5'-3')	Anti-sense primer (5'-3')	Tm(°C)	Amplicon size(bp)

***Hsp72***	NM_010479	5'ACGGCATCTTCGAGGTGAA 3'	5' TGTTCTGGCTGATGTCCTTCT 3'	50	129
***Hprt1***	NM_013556	5'TCCTCCTCAGACCGCTTTT3'	5'AGGTATACAAAACAAATCTAGGTCAT3'	48	118


Real-time PCR was conducted in a real-time cycler (Applied Biosystems 7500, CA, USA). To confirm the specificity and integrity of the PCR products, melting curve analyses were performed for all real-time PCR reactions. Standard curves were generated using serial dilutions of cDNAs. The cDNA of each sample was used as template for the preliminary PCR by AmpliTaq Gold PCR Master Mix according to the manufacturer’s instruction. Reactions were performed in a final volume of 50 μl. The firstround PCR mix contained 2 μl specific primer mix (300 nM), 25 μl master mix, 5 μl cDNA and 18 μl sterile water.

The first-round PCR was performed in a thermal cycler (Applied Biosystem 2720, California and USA), by incubation at 95˚C for 5 minutes, followed by 30 cycles of 95˚C for 15 seconds, specific Tm for every gene for 15 seconds (Table 1), and 72˚C for 60 seconds, and a final extension at 72˚C for 7 minutes. The PCR products were separated on 3% agarose gel (pure Nusieve GTC Agarose, Rockland, USA).

Real time PCR was conducted for cDNA and standards in triplicates with two no-template controls (NTC). Reactions (25 μl) contained 12.5 μl Platinum® SYBR® green qPCR super mix-UDG, 0.5 μl Rox Reference dye, 0.5 μl primer mix (sense and antisense primers, 300 nM each), 6.5 μl autoclaved distilled water and 5 μl of cDNA in every well.

Cycling parameters were 50˚C for 2 minutes (UDG incubation), 95˚C for 2 minutes, followed by 50 cycles of 95˚C for 15 seconds and 60˚C for 30 seconds. The melting curve was analyzed at 95˚C for 15 sand temperature lowered to 60˚C for 15 seconds. Every experiment was repeated three times.

The data were analyzed with the integrated ABI 7500-V2.0.1 software (Applied Biosystem, California, USA) and were normalized with Hprt1 within the log linear phase of the amplification curve using the comparative Ct method (cycle threshold). The relative expression ratio (R) of *Hsp72* was estimated based on a ΔCt formula (31-33). PCR efficiencies (32,33) of the genes ranged between 1.98-2.0. ΔCt was the difference between the Ct values of controls and samples.

### Statistical analysis

One-way analysis of variance (ANOVA) was performed on the average percentages of survived, cleaved embryos, blastocyst formation and relative amount of *Hsp72* mRNA in control, vit1 and vit_2_ groups. Following the analysis of variance, mean values were compared. The level of significance was set at less than 0.05 and least significant difference (LSD) test was used to compare treatments.

## Results

### Developmental competence of 2-cell embryos following vitrification

In total, 195 in-vivo embryos at 2-cell stage were evaluated for survival, cleavage and blastocyst rates in control, vit1 and vit_2_ groups. The survival rates of vitrified and control groups are summarized in table 2, with no difference between vitrified groups and significantly lower than control (p< 0.05).

**Table 2:** The survival rates of 2-cell embryos in control and vitrified groups


Groups	Concentration of cryoprotectans	No. of total embryos	No. of survived embryos	Mean of survival rate (%)	Standard deviation

**control**	0%	76	73	95.8^a^	0.06
**vit_1_**	15% (7.5% EG + 7.5% DMSO)	55	41	75.3^b^	0.13
**vit_2_**	30% (15% EG + 15% DMSO)	64	45	68.6^b^	0.07


Control; Non-vitrified group, DMSO; Dimethyl sulfoxide and EG; Ethylene glycol, a and b indicate significant difference between control with vitrified groups (p<0.01). Every experiment was repeated three times.

**Fig 1:**
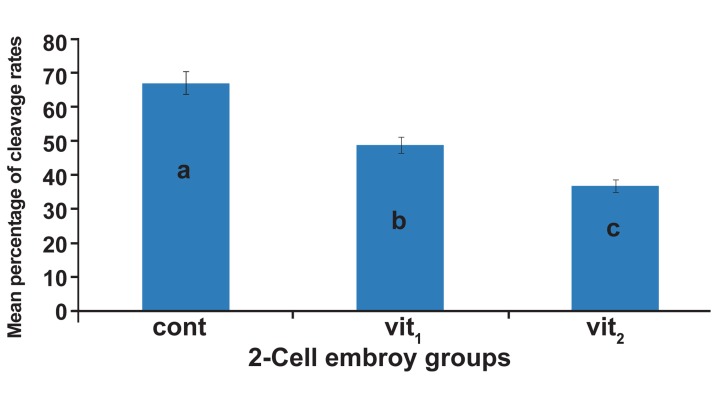
Mean of cleavage rates of 2-cell embryos (to morula) in three groups, cont; control (non-vitrified) group, vit1 ; vitrification with 7.5% DMSO and 7.5% EG, vit_2_ ; vitrification with 15% DMSO and 15% EG. a, b and c indicate the significant differences among control, vit1 and vit_2_ (p<0.01).

The cleavage rates of embryos (2-cell to morula) in all groups are shown in figure 1. The cleavage rate in control (67.1% ± 1.6) was significantly higher than vit1 (48.8% ± 0.9). Furthermore, the cleavage rate in vit1 was significantly higher than vit_2_ (36.8% ± 1.2) groups (p<0.05).

**Fig 2:**
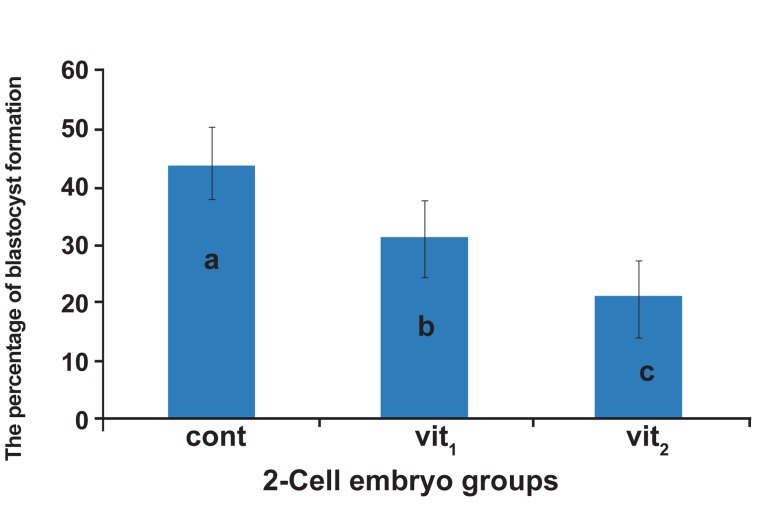
The percentages of blastocyst formation of 2-cell embryos in three groups, cont; control (non-vitrified) group, vit1 ; vitrification with 7.5% DMSO and 7.5% EG, vit_2_ ; vitrification with 15% DMSO and 15% EG. a, b and c indicate the significant differences among control, vit1 and vit_2_ (p<0.05).

The percentages of blastocyst formation in the control, vit1 and vit_2_ groups were 43.8 ± 1.4, 31.7 ± 0.9 and 21.1 ± 0.8, respectively (Fig 2). The differences among the means of control, vit1 and vit_2_ were significant (p<0.05).

### Expression of Hsp72 mRNA

**Fig 3:**
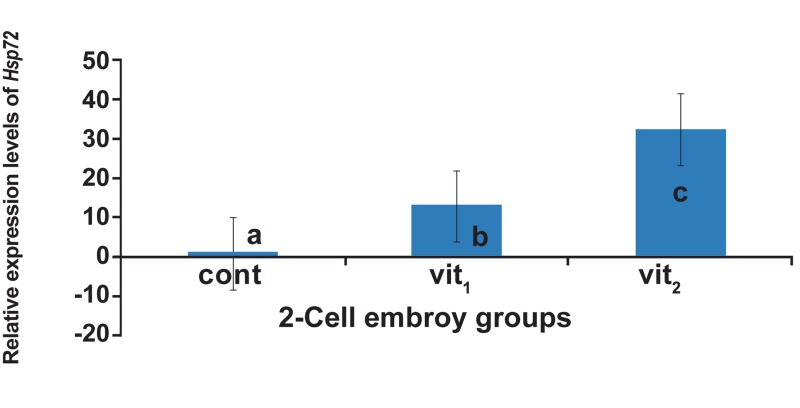
The relative quantification of *Hsp72* after normalization by Hprt1 in 2-cell embryo groups, cont; control (nonvitrified) group, vit1 ; vitrification with 7.5% DMSO and 7.5% EG, vit_2_ ; vitrification with 15% DMSO and 15% EG. a, b and c indicate the significant differences among control, vit1 and vit_2_ (p<0.05).

The effect of different concentrations of cryoprotectants on the expression of *Hsp72* in 2-cell embryos was analyzed with nqPCR and the data were normalized against Hprt1. *Hsp72* was significantly up-regulated, 12.9 fold in vit1 and 32.4 fold in vit_2_, when compared to the control group (p<0.05, Fig 3). Moreover, the normalized relative expression ratio of *Hsp72* in vit_2_ was significantly higher than vit1 (p<0.05).

**Fig 4:**
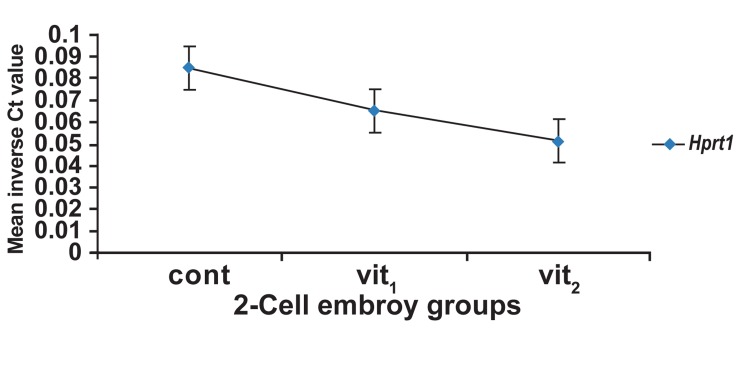
Mean inverse Ct values of Hprt1 as the relevant abundance of transcript 2-cell embryo groups, Ct; threshold cycle, cont; control (non-vitrified) group, vit1; vitrification with 7.5% DMSO and 7.5% EG, vit_2_ ; vitrification with 15% DMSO and 15% EG. Bars are indicative of having no significant difference.

Mean inverse Ct values of Hprt1 had no significant differences between vitrified and control groups (p>0.05, Fig 4).

## Discussion

Mouse embryos can be cryopreserved efficiently at a wide range of developmental stages, 2-cell, 8-cell, or the morula stage (25,34). During the previous years, the success rates of vitrification have been improved by speeding up the cooling rates of cells via minimizing the sample size (vitrification solution and embryos). This increase effectively prohibits the ice crystal formation (1,25,35). Despite the fact that vitrification has proved to be useful in many aspects of cryobiology and fertility restoration, possible molecular consequences of vitrification are yet to be addressed properly. Initially, this can be ascertained through detailed molecular studies of genes that are directly involved in response to temperature change and stress response (36,37).

Association of Hsp70, Hsp27 and Hsp90 subfamilies have been demonstrated in the protection against apoptosis induced by a variety of stimuli such as heat shock, reactive oxygen species and cytoskeletal perturbation (38-40). Amongst the family of Hsp, *Hsp72* is reported to be expressed at 2-cell embryos. For this reason, *Hsp72* was considered as are presentative of the genes that maybe affected during vitrification with a variety of cryoprotectant concentrations. A concentration of a cryoprotectant is considered suitable when the expression pattern and morphological features of the fresh 2-cell embryos can be replicated as closeas possible. Indeed, this means that the cryoprotectant has had minimal effects on the cells.

Here, the previously proposed concentration of cryoprotectants (15% DMSO + 15% EG) was compared with the reduced concentration (7.5% DMSO +7.5% EG) in cryotop vitrification method. Ultimately, their effects on survival and developmental rates and on the expression of *Hsp72* were compared with the control group (non-vitrification).

The results of the present study demonstrated that the survival rates were the same for both vitrification treatments, but the cleavage and blastocyst formation rates in vit1 (our proposed concentration) were significantly higher than vit_2_ for 2-cell mouse embryo. This may suggest reduced vitrification solution toxicity for vit1 as opposed to vit_2_. Moreover, the survival and development rates of vitrified embryos were significantly lower than non-vitrified embryos. This might be due to the vitrification-thawing treatment of the embryos at an early stage of development and further be the result of poorly developed stress response mechanisms. In contrast to our results, vitrification of human oocytes and embryos had no negative effect on survival and developmental rates (14,17,18). These dissimilar outcomes can be explained by the differences that are present between mice and human embryonic cells such as size and shape of the cells and membrane permeability (5).

Two other genes that were previously reported to be expressed in 2-cell embryos (41,42) were also considered for transcript analysis,Gja1 (Connexin 43), a gap junction gene (43-45), and Ped genes, a gene family regulating the rate of preimplantation embryonic development and subsequent embryo survival (46-48). However, our attempts to detect any expression of these genes at this stage failed (data not shown). Transcript analysis of *Hsp72* showed an upregulation in vitrified groups when compared to the control group, similar to the previous results following other vitrification methods (25,49). Furthermore, the relative quantification of *Hsp72* in vit1 was significantly lower than vit_2_ and closer to the fresh 2-cell embryos. The fact that Hsps play a protective role during imposed stresses to the cells, suppressing several forms of cell death, including apoptosis (50) may suggest that vit1 treatment had a lesser impact on the overall well-being of the cell. In general, it can be said that 2-cell mouse embryos have experienced thermal stress during vitrification steps, but the concentrated cryoprotectants causes a pronounced stress to the embryos.

## Conclusion

Our developmental data show that cryotopvitrification with 7.5% EG and 7.5% DMSO was more efficient than that with 15% EG and 15% DMSO. Although vit1 treatment had lower survival and developmental rates compared to the control group, it demonstrated better stability compared with vit_2_ based on the *Hsp72* transcript analysis, supporting developmental data.
